# *Chlamydia* and mitochondria - an unfragmented relationship

**DOI:** 10.15698/mic2017.07.582

**Published:** 2017-06-14

**Authors:** Suvagata R. Chowdhury, Thomas Rudel

**Affiliations:** 1 Department of Microbiology, University of Wuerzburg, Am Hubland, 97074 Wuerzburg, Germany.

**Keywords:** Chlamydia, mitochondria, metabolism, miRNA

## Abstract

Presence of pathogens within a eukaryotic cell is apt to generate stress. Such stress eventually leads to host defense responses, which includes, but is not limited to, apoptosis induction and subsequent destruction of the host cell and the pathogen. Obligate intracellular pathogens such as *Chlamydia trachomatis* are dependent on the survival of the host cell owing to their unique replication niche within a membrane-bound inclusion. Furthermore, being energy parasites, chlamydial development is strictly dependent on the host metabolism. Over the past decade the role of the small non-coding RNAs called microRNAs (miRNAs) have come into focus with respect to the regulation of apoptotic signaling, metabolic homeostasis and bacterial pathogenesis. Effect of *Chlamydia* infection on the host miRNA profile was hitherto unknown. In our recent work we demonstrated that *Chlamydia* infection induces and requires an upregulation of the host miRNA, miR-30c-5p (miR-30c) to ameliorate infection induced stress on the host mitochondrial architecture and hinders induction of apoptosis.

The obligate intracellular pathogen *Chlamydia* depends on metabolites provided by the host cell for their growth and development. To this end the survival and maintenance of the basal metabolic homeostasis of the *Chlamydia*-infected host cell are paramount for the completion of the intracellular life cycle of the pathogen. The anti-apoptotic strategies employed by *Chlamydia* are well investigated and are found to be efficient in blocking host cell death induced by a wide range of external and internal pro-cell death stimuli. *Chlamydia* has been known to inhibit the premature apoptosis of the host cell via the stabilization of host anti-apoptotic proteins such as Mcl-1. Over the last decade, the discovery of small non-coding RNA based control over cellular signaling pathways has increasingly pointed out the high degree of regulation exerted by miRNAs over specific signaling cascades. Unsurprisingly, a whole group of diverse miRNAs, termed as the Apoptomirs, has been shown to regulate the apoptotic signaling cascades by targeting a variety of pro- or anti- apoptotic proteins.

Several groups have shown that bacterial, parasitic and viral infections can trigger changes in the host miRNA profile. While it has been known for some time that several DNA viruses encode and utilize their own miRNAs to facilitate infection and pathogenesis, it has also been shown that they can regulate the expression profile of several host miRNAs to aid in the infection process. On the other hand, effort to understand and study the effect of bacterial infection on the host miRNA expression has been gaining considerable momentum. The changes in the host miRNA expression profile upon *Helicobacter pylori* and *Salmonella enterica* infection are well explored. Our aim was to investigate the hitherto uncharted host miRNA expression landscape upon *Chlamydia * infection. Upon performing a miRNA deep sequencing screen of *Chlamydia* infected Human Umbilical Vascular Endothelial Cells (HUVECs) we discovered that several miRNAs known to be involved in apoptotic signalling exhibited changes in expression. This was unsurprising in the light of the well-established anti-apoptotic nature of *Chlamydia*. However, we chose to focus on one of the lesser-known miRNAs, miR-30c-5p, which was significantly upregulated upon *Chlamydia* infection in the screen.

Members of the miR-30c family have been shown to target the tumour suppressor protein p53, which has also been demonstrated to be downregulated upon *Chlamydia *infection. While we have previously determined that the immediate downregulation of p53 upon *Chlamydia* infection is a result of proteasomal degradation of the protein mediated by the PI3K-Akt dependent activation of HDM2, our recent work shows that the miR-30c dependent suppression of p53 translation occurs close to 20 hours post infection and appears to be a stable means to ensure low levels of p53. Loss of p53 is important for *Chlamydia* development since DNA damage mediated activation of p53 suppresses the pentose phosphate pathway necessary for *Chlamydia* growth. Additionally, in our recent work, we observed that eradication of miR-30c expression using inducible miRNA sponges or miRNA inhibitors led to p53 stabilization, irreversible fragmentation of the mitochondrial network and severely hindered *Chlamydia* growth. The effect of miR-30c modulation on the mitochondrial architecture has been previously noted in other studies since p53 is a transcriptional regulation of the major mitochondrial fission regulator Drp1. Stabilization of p53, either by artificial over expression or by inhibition of miR-30c results in upregulation of Drp1 and promotes mitochondrial fragmentation.

We speculated that besides the obvious effect of p53 stabilization on *Chlamydia* growth, it might be possible that the mitochondrial architecture may have a significant role on chlamydial pathogenesis. To this end we artificially overexpressed wild type and catalytically inactive Drp1 in p53 null H1299 p53-/- cells and discovered that wild type Drp1 expression induces massive mitochondrial fragmentation even in absence of p53 and prevents establishment of *Chlamydia* infection. We used SR-SIM to quantify Drp1 aggregate counts and mitochondrial co-localization and found that both were reduced upon *Chlamydia* infection. The infected cells also failed to exhibit any increase in either parameter upon treatment with known fission inducing agents such as H_2_O_2_. These results point out that the miR-30c-mediated p53 downregulation not only functions to promote the pentose phosphate pathway activation but also to preserve the mitochondrial architecture by reducing Drp1 levels. In accordance with the infection-induced Drp1 downregulation, quantitative microscopy using custom designed MACRO scripts revealed that *Chlamydia* infection promoted mitochondrial elongation and altered mitochondrial dynamics in HUVECs and primary epithelial cells cultured from biopsies of the human fallopian tube fimbriae tissue (hFIMB cells; Figure 1A and 1B).

**Figure 1:**
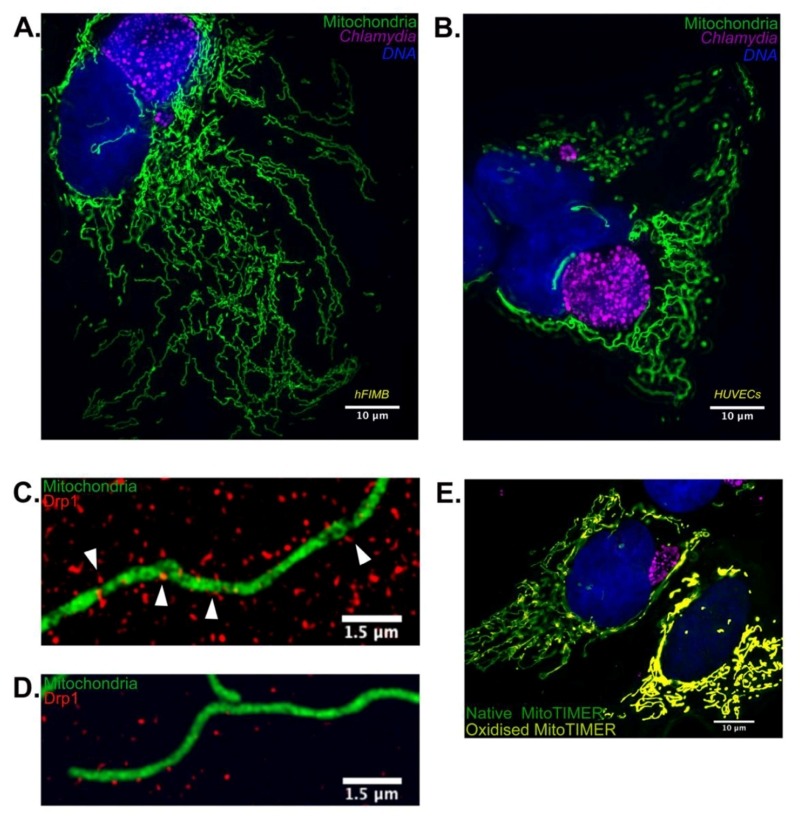
FIGURE 1: Chlamydia infection alters mitochondrial morphology in primary cells by inhibition of Drp1 expression. **(A)** 36 hours of *Chlamydia* infection in hFIMB cells lead to an abnormal increase in mitochondrial fragment length while preventing H^2^O_2_ mediated fragmentation. The sample was treated with 1μM H_2_O_2_ for 1 hour. **(B)** In this panel both the HUVECs have been treated with CoCl_2_ for 6 hours, however, only the cell harboring a very early stage *Chlamydia* inclusion (8 hours) undergoes severe mitochondrial fragmentation. Advanced stages of *Chlamydia* infection (36 hours; right most cell) grants the host cell mitochondria protection against extrinsic pro-fragmentation stress mediated by CoCl_2_. Fragments of mitochondria from **(C)** non-infected and **(D)** *Chlamydia*-infected HUVECs decorated with Drp1 aggregates. The white arrows in (C) indicate successful Drp1 aggregate association with the mitochondria and initiation of fission. ** (E)** HUVECs transfected with MitoTIMER and infected with *Chlamydia* for 20 hours. The non-infected cell (right most) exhibits a drastic increase in the red form (oxidized form) of the MitoTIMER protein along with a decrease in mitochondrial fragment length.

Another interesting observation of our recent publication was that *Chlamydia* itself generates stress within the mitochondrial matrix. *Chlamydia* has been shown to promote transient production of ROS necessary for the establishment of infection and growth. Under normal circumstances, such stress would be sufficient to promote mitochondrial fragmentation and subsequent degradation of the mitochondrial fragments. Using the fluorescent Timer protein targeted to mitochondrial matrix, which alters its fluorescent properties upon encountering ROS, we found that *Chlamydia* reversibly stresses the mitochondrial matrix. However, in absence of Drp1-guided mitochondrial fission, the mitochondrial architecture fails to fragment and is protected from both intrinsic and extrinsic pro-fragmentation stimuli (Figure 1E). This observation has multifaceted implications.

Firstly, we observed that the reversibility of mitochondrial stress and inhibition of mitochondrial fission was limited only to the cells that harbored *Chlamydia* inclusions and not the uninfected cell within the same culture vessel. Most of the uninfected cells exhibit stressed and fragmented mitochondria, which led us to rationalize that the protective effect of *Chlamydia* on the host mitochondrial network is possibly a result of the miR-30c-mediated p53 and Drp1 suppression within infected cells. Secondly, it has been observed that mitochondrial particles undergo elongation upon facing nutrient stress. Such elongation spares them from mitophagy-mediated degradation. However, in cases of nutrient-mediated stress, the mitochondrial elongation appears to be a result of mitofusin-induced increase in fusion events. Using live cell imaging we found that while mitochondrial fission events were markedly absent in *Chlamydia*-infected cells, the number of fusion events remained more or less equal to those of control or uninfected cells. While the net result in both cases produce elongated mitochondria, it raises the question if mitochondrial fusion is affected upon infection. Finally, elongated mitochondria have been shown to exhibit higher capacity of ATP synthesis and these elongated mitochondria are specifically spared from degradation under conditions of starvation-induced autophagy. *Chlamydia* is well known as an “energy parasite” and within primary cells such as HUVECs, the pathogen is heavily dependent upon the mitochondrial supply of ATP, the main source of ATP synthesis. To confirm this idea, we generated HeLa cells with inducible knock down of F1β subunit of the mitochondrial F_1_F_0_-ATPase. While knock down of F1β subunit in HeLa cells grown in normal media with glucose had no effect on the cellular ATP levels, inhibition of the cytoplasmic glycolytic pathway with galactose in conjunction with the knock down of F1β subunit resulted in depletion of ATP levels and severely hindered chlamydial growth.

The parasitic nature of *Chlamydia* has been discussed ever since it was observed that the pathogen lacks key enzymes necessary for production of essential metabolites and that its genome encodes (ADP)/ATP translocases. Our current work demonstrates that, not only does *Chlamydia* require the mitochondrial source of ATP within primary cells but also employs a very cunning strategy that protects the host mitochondrial architecture form the intrinsic stresses generated by the infection process itself.

